# PalKCH1 and PalKCH2 are required for tension wood formation and gelatinous layer deposition in *Populus*

**DOI:** 10.3389/fpls.2026.1942251

**Published:** 2026-09-21

**Authors:** Jianglong Li, Kunjing Qu, Rui Wang, Jie Zhang, Ningning Zhou

**Affiliations:** State Key Laboratory of Herbage Improvement and Grassland Agro-ecosystems, College of Ecology, Lanzhou University, Lanzhou, Gansu, China

**Keywords:** cell wall remodeling, gelatinous layer, gravitropism, PalKCH1/2 genes, tension wood

## Abstract

Tension wood (TW) is a graviresponsive xylem tissue exclusive to angiosperm trees that counteracts gravitational stress. Its hallmark is a gelatinous layer (G-layer) in fiber secondary walls, rich in cellulose and low in lignin. Although kinesins mediate microtubule–microfilament crosstalk, their role in G-layer biogenesis during gravistimulation is unclear in woody angiosperms. We studied two kinesin paralogs, *PalKCH1* and *PalKCH2*, in *Populus alba* var. *pyramidalis* during TW development. Both belong to the Group I KCH clade, possessing a kinesin motor domain and a calponin-homology (CH) domain. Multi-omic profiling showed preferential expression in xylem fibers and strong gravistimulation-induced transcription. CRISPR/Cas9 single and double knockout mutants showed normal xylem under upright growth, but responded to gravitational stress with reduced stem bending, narrower TW regions, and fewer G-layer fibers; double mutants were more affected, suggesting dosage dependence. Chemical assays showed that mutants retained the cellulose-rich, lignin-poor TW signature, but the changes were attenuated compared to wild-type. Transcriptomic analysis of double mutants revealed suppression of genes involved in microtubule dynamics, vesicular trafficking, cell wall remodeling, reduced *PalWOX4A* expression, and repressed cell cycle pathways. Thus, *PalKCH1* and *PalKCH2* are required for TW formation, likely via cytoskeletal signaling, and represent high-value targets for improving wood quality in *Populus*.

## Introduction

In complex terrestrial environments, perennial woody plants maintain upright growth through the negative gravitropism of their stems (Morita, 2010). When a stem is displaced from the vertical by external forces or its own weight, mature, secondarily thickened stems can no longer reorient themselves through cell elongation alone. Consequently, woody plants that possess a vascular cambium and produce secondary xylem have evolved a specialized adaptive trait—the formation of reaction wood (Groover, 2016). In gymnosperms, compression wood develops on the lower side of a tilted stem, whereas in woody angiosperms such as *Populus*, tension wood (TW) forms on the upper side. The axial contractile force generated by TW fibers then pulls the stem back toward the vertical (Goswami et al., 2008; Groover, 2016).

The most distinct cytological feature of TW fibers is the deposition of a specialized gelatinous layer (G-layer) on the inner side of the cell wall. This layer is primarily composed of cellulose, with a negligible lignin content, and the cellulose microfibrils within it are aligned almost parallel to the longitudinal axis of the cell. These G-layers provide the structural basis for the axial contractile force that drives the gravitropic correction of stems (Mellerowicz and Gorshkova, 2012). Elucidating the molecular mechanisms that control the ordered, directional deposition of cellulose in the G-layer is therefore essential for understanding how trees adapt to gravitational stress and regulate wood formation.

The ordered deposition of G-layer cellulose relies on the dynamic coordination between actin microfilaments and cortical microtubules. Specifically, microfilaments provide the tracks for secretory vesicle transport, facilitating the targeted delivery of cellulose synthase complexes (CSCs) to the plasma membrane (Zhang et al., 2019; Zhang and Staiger, 2021; Liu et al., 2023). In parallel, cortical microtubules guide CSC trajectories within the membrane, thereby dictating the orientation of cellulose microfibrils (Zhao et al., 2025). The necessity of this interplay is underscored by findings in cotton fibers, where microfilament disruption triggers the disorganization of cortical microtubule arrays (Seagull, 1990), suggesting a functional coupling that is critical for directional cell wall assembly.

The plant-specific calponin homology (CH) domain-containing kinesin (KCH) is a strong candidate for mediating the aforementioned cytoskeletal coupling. Cross-species studies indicate that the KCH-mediated bridging of dual cytoskeletal systems is highly conserved, although functional differentiation has occurred throughout land plant evolution. In the basal land plant *Physcomitrium patens*, PpKCHb localizes to microtubules, and its deficiency impairs gravity-induced actin filament rearrangement, resulting in gravitropism defects (Li et al., 2021); in the monocot *Oryza sativa*, OsKCH1 functions as a cytoskeletal bridge by binding both actin filaments and cortical microtubules, thereby facilitating nuclear positioning during interphase (Frey et al., 2009); in the dicot fiber model *Gossypium hirsutum*, GhKCH1 localizes to cortical microtubules and binds actin filaments, likely mediating actin transport to support rapid fiber elongation (Preuss et al., 2004). Furthermore, in the dicot *Arabidopsis thaliana*, the AtKCH7 protein, encoded by the *GhKCH1* ortholog *AtKATD*, regulates root gravitropism by modulating auxin distribution through actin bundle formation (Niu et al., 2025). These findings suggest that while KCH family functions have undergone significant diversification, their fundamental role in physically bridging actin filaments and cortical microtubules remains highly conserved. Nevertheless, existing research has primarily focused on KCH in the context of primary growth and root gravitropism; there is currently no evidence regarding the involvement of KCH in secondary cell wall formation or cellulose deposition within the G-layer of TW in woody plants.

TW represents a specialized secondary xylem program initiated in response to gravity stress, and G-layer development relies heavily on the synergistic interaction between microfilaments and microtubules. We hypothesize that the KCH family in *Populus* acts as a key bridge linking these two cytoskeletal systems, thereby regulating G-layer formation in TW. Protein sequence alignment shows that PalKCH1 and PalKCH2 from *Populus alba* var. *pyramidalis* are highly homologous to cotton GhKCH1, both possessing conserved CH domains and kinesin motor domains. Multi-omics analysis in *Populus trichocarpa* further reveals that their orthologs, *PtrKCH1/2*, are specifically expressed in xylem fiber cells and strongly upregulated during TW induction, exhibiting spatiotemporal expression patterns tightly correlated with G-layer development. Consequently, this study proposes the core hypothesis that during TW development, PalKCH1/2 proteins mediate dynamic cytoskeletal remodeling by physically bridging microfilaments and cortical microtubules, functioning as critical regulators that orchestrate the ordered deposition of G-layer cellulose microfibrils.

To test this hypothesis, we used *Populus alba* var. *pyramidalis* as a model. We generated *PalKCH1/2* double-knockout mutants via CRISPR/Cas9 and combined phenotypic characterization of TW, cell wall compositional analysis, and RNA-sequencing to elucidate the molecular function of *PalKCH* genes in TW development. These results advance our mechanistic understanding of gravitropic adaptation in woody plants and provide a foundation for genetically improving wood fiber traits.

## Materials and methods

### Genomic data acquisition

The protein sequences and genome annotation files for *Physcomitrium patens* (Pp) and *Oryza sativa* (Os) were retrieved from the NCBI database. The genome, protein, and annotation files for *Populus alba* var. *pyramidalis* (Pal) were obtained from the Genome Warehouse at the China National Center for Bioinformation (CNCB-NGDC) (Ma et al., 2025; CNCB–NGDC Members and Partners et al., 2026). The genome, protein, and annotation files for *Arabidopsis thaliana* (At) and *Populus trichocarpa* (Ptr) were downloaded from Phytozome v14. Detailed accession numbers and version information for all species are provided in **Supplementary Table S1**.

### Identification of *PalKCH* genes in *Populus alba* var. *pyramidalis*

A BLASTP search was conducted against the *Populus alba* var. *pyramidalis* genome with all seven *Arabidopsis thaliana* KCH proteins as queries (Richardson et al., 2006) using TBtools-II (Chen et al., 2023) to identify putative KCH orthologs. Candidate sequences were subsequently validated for the presence of conserved domains using the InterPro and NCBI Conserved Domain Database (CDD) tools. Candidates lacking either the CH domain or the motor domain were excluded from further analysis.

### Phylogenetic analysis and gene structural characterization

Multiple sequence alignment (MSA) was performed on the amino acid sequences encoded by the 11 *PalKCH* genes identified in this study, along with 7 *AtKCH* genes (Richardson et al., 2006), 9 *OsKCH* genes (Tseng et al., 2018), and 4 *PpKCH* genes (Yamada and Goshima, 2018) reported in the literature, as well as the representative cotton *GhKCH1* gene (Preuss et al., 2004) and *GhKCH2* gene (Xu et al., 2009), using MAFFT (Katoh and Standley, 2013). Detailed amino acid sequences are provided in **Supplementary Table S2**. The resulting alignments were refined using trimAl (Capella-Gutiérrez et al., 2009). Subsequently, a phylogenetic tree was constructed using IQ-TREE3 (Wong et al., 2026). The tree was visualized and refined using the ChiPlot platform (Xie et al., 2023). Conserved domain analysis was conducted for all sequences using the NCBI Batch CD-Search tool. The phylogenetic tree, protein domain architectures, and gene structural features were integrated and visualized using the “Gene Structure View (Advanced)” function in TBtools-II (Chen et al., 2023).

### Bioinformatics analysis of *PtrKCH* gene responses to TW formation

We downloaded the RNA-seq dataset PRJNA706666 (Yu et al., 2022) from the NCBI SRA database. Raw sequencing data in FASTQ format were processed using fastp (Chen et al., 2018) for adapter trimming and filtering of low-quality bases. The filtering parameters were set as follows: minimum phred quality score of 20 (-q 20), minimum read length threshold of 36 bp (-l 36), automated adapter detection for paired-end data (--detect_adapter_for_pe), and sliding window quality trimming at both 5’ and 3’ ends (--cut_front --cut_tail). High-quality clean reads were mapped to the *Populus trichocarpa* reference genome with the corresponding gene annotation file using HISAT2 (Kim et al., 2019). The resulting SAM files were converted to BAM format via SAMtools (Li et al., 2009), followed by transcript assembly and raw count quantification using StringTie (Pertea et al., 2015). Detailed metadata, sequencing depth, mapping rates, and reference genome/annotation resources are compiled in **Supplementary Table S3**.

The GSE153797 dataset (Liu et al., 2021) was also downloaded from the NCBI GEO database for cross-validation purposes. In the R environment, we utilized the GenomicFeatures package (Lawrence et al., 2013) and the *Populus trichocarpa* GFF3 annotation file to calculate the total non-overlapping exon length for the longest transcript of each gene. These values served as the effective gene lengths. Finally, the raw count matrix was normalized using the TPM (Transcripts Per Million) formula (Li and Dewey, 2011) to obtain the standardized TPM expression matrix.

The RNA-seq data matrix derived from laser capture microdissection (LCM) was obtained from the GEO database, accession number GSE295131 (Hsieh et al., 2025). Differential expression analysis was performed using TBtools-II (Chen et al., 2023). Differentially expressed genes (DEGs) were defined as genes with *P*_adj_ < 0.05 and |log_2_(fold change)| > 1.

### Single-cell transcriptomic analysis

Single-cell transcriptomic data were obtained from the GEO database (accession number GSE267159; Hsieh et al., 2025). Data processing and analysis were performed in R. The Seurat package (Hao et al., 2024) was utilized for preprocessing; specifically, the raw expression matrix was imported via the Read10X_h5 function, and a Seurat object was constructed using CreateSeuratObject, with standard filtering criteria applied to remove low-quality cells and empty droplets. Cell cluster annotations provided by the original study were integrated into the Seurat object metadata via barcode matching, serving as the basis for subsequent analyses. Data normalization was performed using the NormalizeData function (via the LogNormalize method) to account for differences in library depth. Finally, the AverageExpression function was used to calculate the mean expression levels of *PtrKCH* genes across distinct cell clusters.

### Plant materials and treatments

*Populus alba* var. *pyramidalis* wild-type (WT) plants were used in this study. Plants were grown in a controlled environment greenhouse at 22–25 °C, 50% ± 10% relative humidity, under a 16 h light/8 h dark photoperiod. After two months of growth, individuals of uniform vigor were selected and divided into control and TW induction groups, with three biological replicates per group. To induce TW formation, plants in the treatment group were placed horizontally for 14 days, while control plants were maintained in an upright position. Upon completion of the treatment, xylem samples were collected for tissue-specific localization analysis of *PalKCH* genes and experimental validation of their response to gravity-induced TW formation.

### Selection of reference genes

For tissue-specific expression analysis, reference genes and their corresponding primer sequences were adopted from published literature (Tong et al., 2021). Regarding the TW verification experiments, candidate reference genes were selected based on publicly available transcriptome data of TW formation in *Populus trichocarpa*. Initially, housekeeping genes exhibiting high expression levels and minimal fluctuations between normal wood (NW) and TW were selected as candidates. These five candidates were then evaluated in pooled samples to assess their expression stability (**Supplementary Figures S1A–C**). Subsequently, *PalSF* (*Palv1_08791*) was identified as the optimal reference gene and subjected to secondary validation across all biological replicates (n = 3). The criterion for validation was a difference in threshold cycle values (|ΔCt values| < 0.5) between the NW and TW groups (**Supplementary Figure S1D**). Consequently, *PalSF* was established as the internal control for the TW experimental group.

### RT-qPCR analysis

Gene expression levels were quantified using the 2 × PerfectStart^®^ Green qPCR SuperMix (TRAN, Beijing, China) on a LightCycler 96 real-time PCR system (Roche, Basel, Switzerland). The *PalSF* gene was employed as the internal control to normalize expression data. Relative expression levels of target genes were calculated using the 2^−△△CT^ method (Schmittgen and Livak, 2008). All RT-qPCR experiments were performed with three biological replicates, each including three technical replicates.

### TW induction and phenotypic analysis

Single and double knockout lines were generated via Agrobacterium-mediated CRISPR/Cas9 transformation (Ma et al., 2015). Genotypes were propagated by stem cuttings and grown under identical greenhouse conditions.

The experimental design comprised two treatment groups: a TW group, induced by maintaining plants in a horizontal position for 14 days, and a control group, in which plants were grown vertically. Each group included WT plants, single-gene knockout lines, and double-gene knockout lines. Tension wood induction followed established protocols (Gerttula et al., 2015).

Stem lifting height and bending angle were determined as described previously (Hu et al., 2024). The initiation point of visible stem curvature was defined as the bending point. Lifting height was calculated as the vertical distance from the stem base to the bending point; this value, along with the total stem length, was used to calculate the degree of stem bending. Each experiment included over four plants per genotype (WT and transgenic lines) and was repeated three times.

Histological analysis was conducted on both TW-treated and control groups. For the TW group, the 8th internode was labeled prior to treatment; after 14 days, the middle section of this pre-labeled internode was sectioned at 50 *μ*m using a vibratome (VT1000S; Leica, Wetzlar, Germany). For control plants, the middle section of the 15th internode was similarly sectioned. All sections were stained with 0.01% (w/v) toluidine blue and examined under an Olympus BX53 microscope (Olympus, Tokyo, Japan). All histological procedures were performed in triplicate.

### Quantification of cell wall components

Lignin and cellulose content in the cell walls of NW and TW samples were quantified according to established protocols (Foster et al., 2010a, 2010b). The study utilized stem segments from *Populus* grown in a greenhouse for 2 months. For the TW-treated plants, the TW tissues were carefully excised to obtain representative samples; for the vertical control plants, whole wood tissues were harvested. All measurements were performed with at least three biological replicates, each including three technical replicates.

### RNA-seq data analysis

This study utilized wild-type and double-knockout plants under both TW induction and vertical growth conditions (n=3 biological replicates per group). For the TW group, tissues characteristic of TW were carefully excised from the stem segments, while corresponding stem regions were sampled from the control group. Actively differentiating xylem cells near the vascular cambium were harvested from both groups using single-edged blades to ensure high-quality transcriptomic profiling. Sequencing was performed by Personalbio (Shanghai, China) using the Illumina BGI T7 platform (paired-end 150 bp). Raw data processing, quality control, and read alignment were performed using fastp and HISAT2, following the pipeline described above. Differential expression analysis was performed using TBtools-II (Chen et al., 2023). DEGs were defined as genes with *P*_adj_ < 0.05 and |log_2_(fold change)| > 1, and reference genome details for all in-house libraries, are summarized in **Supplementary Table S3**.

Following the generation of the transcripts per million (TPM) expression matrix, Pearson correlation coefficients were calculated to assess gene expression consistency across all samples, and hierarchical clustering heatmaps were generated. Furthermore, principal component analysis (PCA) was performed to characterize global transcriptomic differences.

### Gene clustering and functional enrichment analysis

Raw count matrices were subjected to variance-stabilizing transformation (VST) using the DESeq2 package (Love et al., 2014) in R. Genes with low expression (counts < 10 in fewer than six samples) were filtered out. Group means were calculated for four conditions: WT_NW, WT_TW, *palkch1/2*_NW, and *palkch1/2*_TW. The standardized mean expression matrix was imported into the Mfuzz package (Kumar and Futschik, 2007) for fuzzy c-means clustering. The number of clusters was set to 4, the fuzzification parameter was estimated using the mestimate() function, and the random seed was set to 123.

DEGs were identified using the same criteria as described above for the LCM-RNA-seq data matrix. Subsequently, GO and KEGG pathway enrichment analyses were performed on these DEGs and the key gene clusters identified by Mfuzz using the GO and KEGG enrichment tools in TBtools-II (Chen et al., 2023).

### Validation of key genes via RT-qPCR

The expression stability of the preselected reference gene *PalSF* was evaluated using archived transcriptome samples across different genetic backgrounds and experimental conditions. *PalSF* exhibited highly stable expression across the four sample groups (**Supplementary Figure S1E**) and was therefore selected as the internal control for subsequent analysis. RT-qPCR was then performed with *PalSF* as the reference to quantify the key genes identified from the transcriptome data. All experimental procedures followed the “RT-qPCR analysis” section. All assays included at least three biological replicates, each with three technical replicates.

## Results

### Evolutionary conservation and functional prediction of *PalKCH1* and *PalKCH2* in TW

To clarify the evolutionary relationships of the KCH gene family in *Populus alba* var. *pyramidalis*, we constructed a phylogenetic tree using KCH sequences from *Populus alba* var. *pyramidalis*, *Arabidopsis thaliana*, *Oryza sativa*, *Gossypium hirsutum*, and *Physcomitrella patens*. *Physcomitrella patens* sequences were used as the outgroup, reflecting the early divergence of mosses in land plant evolution.

Phylogenetic analysis divided the KCH family into two major clades, Group I and Group II. PalKCH1 and PalKCH2 clustered within subgroup a of Group I, together with GhKCH1, a protein associated with cotton fiber specialization, and AtKCH7 from *Arabidopsis thaliana* (**Figure 1A**). Conserved domain analysis confirmed that all members of this subgroup carry the core CH domain and the kinesin motor domain (**Figure 1A**), indicating a shared domain architecture relevant to cytoskeletal dynamics.

**Figure 1:**
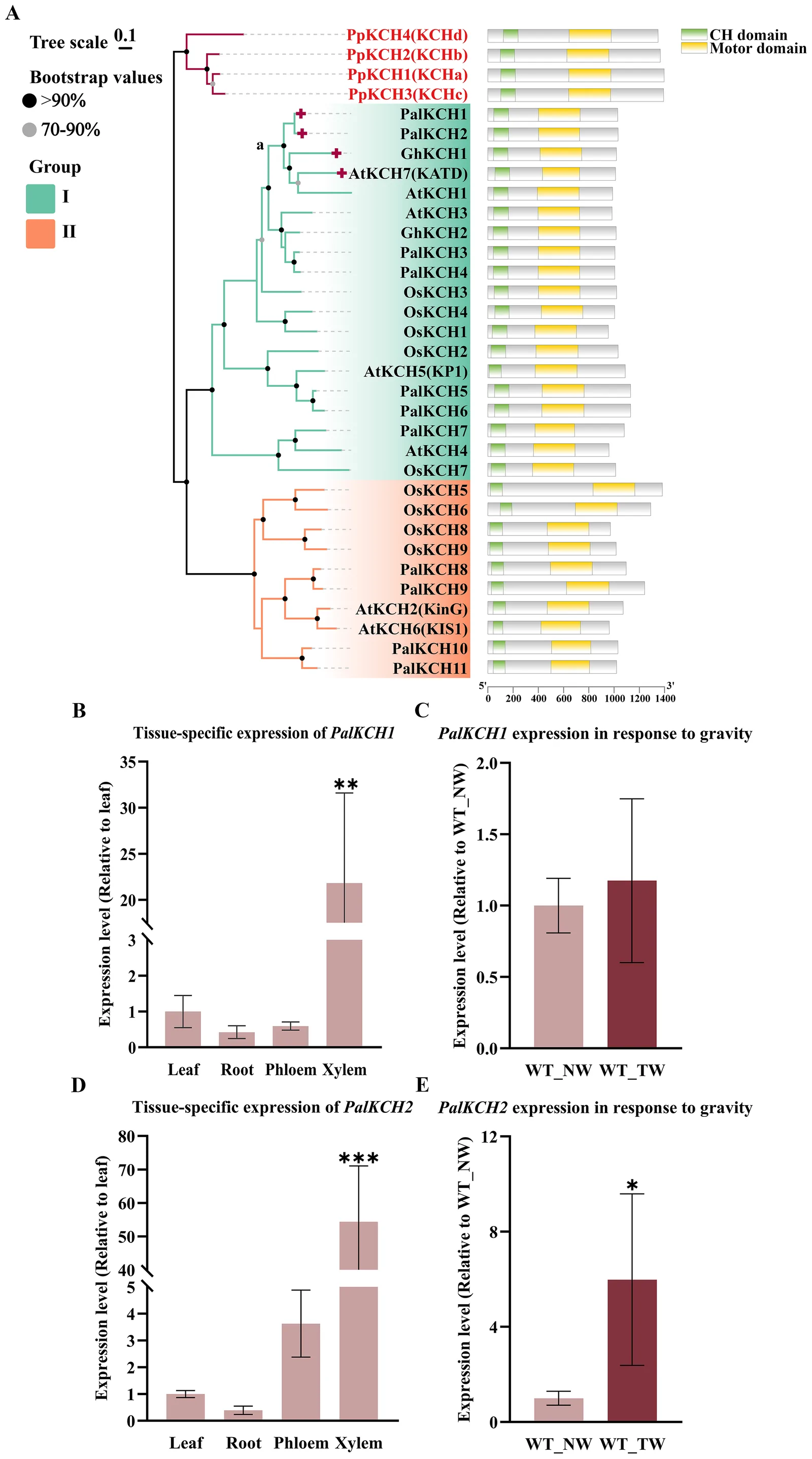
Phylogenetic relationships and expression patterns of the PalKCH gene family. **(A)** Phylogenetic tree of PalKCH proteins, with conserved domain architecture shown alongside. The family is divided into Group I and Group II. Black and gray dots represent bootstrap values >90% and 70–90%, respectively. **(B)** Tissue-specific expression profile of *PalKCH1.* **(C)** Expression of *PalKCH1* in normal wood (NW) and tension wood (TW). **(D)** Tissue-specific expression profile of *PalKCH2*. **(E)** Expression of *PalKCH2* in NW and TW. Data are presented as mean ± SD (n = 3 biological replicates). Asterisks indicate significant differences relative to NW or the indicated control (* *P* < 0.05, ** *P* < 0.01, *** *P* < 0.001; B, D one-way ANOVA with Dunnett’s *post-hoc* test; and C, E unpaired *t*-test with Welch’s correction).

Given the potential role of KCH proteins in secondary wall deposition, we examined the expression patterns of the *PtrKCH* gene family during TW formation using public transcriptomic data and laser capture microdissection (LCM)-based spatiotemporal data from *Populus trichocarpa*. *PtrKCH1*–*PtrKCH4* showed high transcript levels in the xylem and dynamic expression changes during tension wood induction. In particular, PtrKCH1 and PtrKCH2 were significantly upregulated in both macrotemporal datasets (**Supplementary Figures S2A–D**). LCM-based analysis showed that under normal conditions, expression of the *PtrKCH* family in the xylem was minimal. After TW induction, *PtrKCH1*, *PtrKCH2*, and *PtrKCH4* were specifically activated in early fiber cells (regions 1–2), but only *PtrKCH1* remained highly expressed in late-stage fibers (region 3). This pattern points to an early function for *PtrKCH1*, *PtrKCH2*, and *PtrKCH4* during TW fiber development, with *PtrKCH1* potentially acting throughout differentiation.

To determine cell type specificity, we examined single-cell RNA-seq data from *Populus trichocarpa*. *PtrKCH1* and *PtrKCH2* were highly enriched in fiber clusters, whereas *PtrKCH3* and *PtrKCH4* were expressed broadly across multiple cell types (**Supplementary Figures S3A–D**). Together, these independent datasets identify *PtrKCH1* and *PtrKCH2* as the predominant *PtrKCH* genes expressed in developing wood fibers.

We then validated the expression patterns of *PalKCH1*–*PalKCH4* in *Populus alba* var. *pyramidalis* using RT-qPCR. All four *PalKCH* genes showed significantly higher transcript levels in the xylem than in other tissues (**Figures 1B, D**; **Supplementary Figures S4B, D**). *PalKCH2* was significantly upregulated in gravity-induced TW, whereas *PalKCH1* showed only a mild and statistically non-significant increase (**Figures 1C, E**). Although *PalKCH3* and *PalKCH4* also increased in TW, their expression was less specific to developing fibers and less consistent in response to gravitational stimuli. Combined with their fiber-specific expression and close evolutionary relationship, these data suggest that PalKCH1 and PalKCH2 act together during G-layer development in tension wood.

### Phenotypic analysis of xylem development in *PalKCH1/2* double-knockout plants

To investigate the functions of these genes, we generated CRISPR/Cas9-mediated knockout lines, including single mutants (*palkch1#2*, *palkch1#3* for *PalKCH1*; *palkch2#1*, *palkch2#2* for *PalKCH2*) and double mutants (*palkch1/2#5*, *palkch1/2#11*, *palkch1/2#15*) (**Supplementary Figure S5**). Histological sections were prepared from WT and all mutant lines grown under normal conditions (**Supplementary Figure S6B**). Xylem width and area did not differ significantly between mutant lines and WT (**Supplementary Figures S6C, D**), indicating that loss of PalKCH1 and PalKCH2 does not markedly affect xylem development under normal growth conditions.

### Loss of *PalKCH1* and *PalKCH2* leads to defective tension wood phenotypes

To evaluate the roles of PalKCH1 and PalKCH2 during TW formation, we measured stem lift height and curvature in WT, single-mutant, and double-mutant plants at 3, 7, 10, and 14 days after treatment (**Figure 2A**). WT plants showed the greatest stem lifting height and curvature, whereas double-mutant plants showed the weakest response, with single mutants displaying intermediate phenotypes (**Figure 2B**). Two-way ANOVA revealed significant effects of genotype, time, and their interaction (*P* < 0.05), indicating that loss of PalKCH1 and PalKCH2 progressively weakens the stem bending response to gravistimulation.

**Figure 2:**
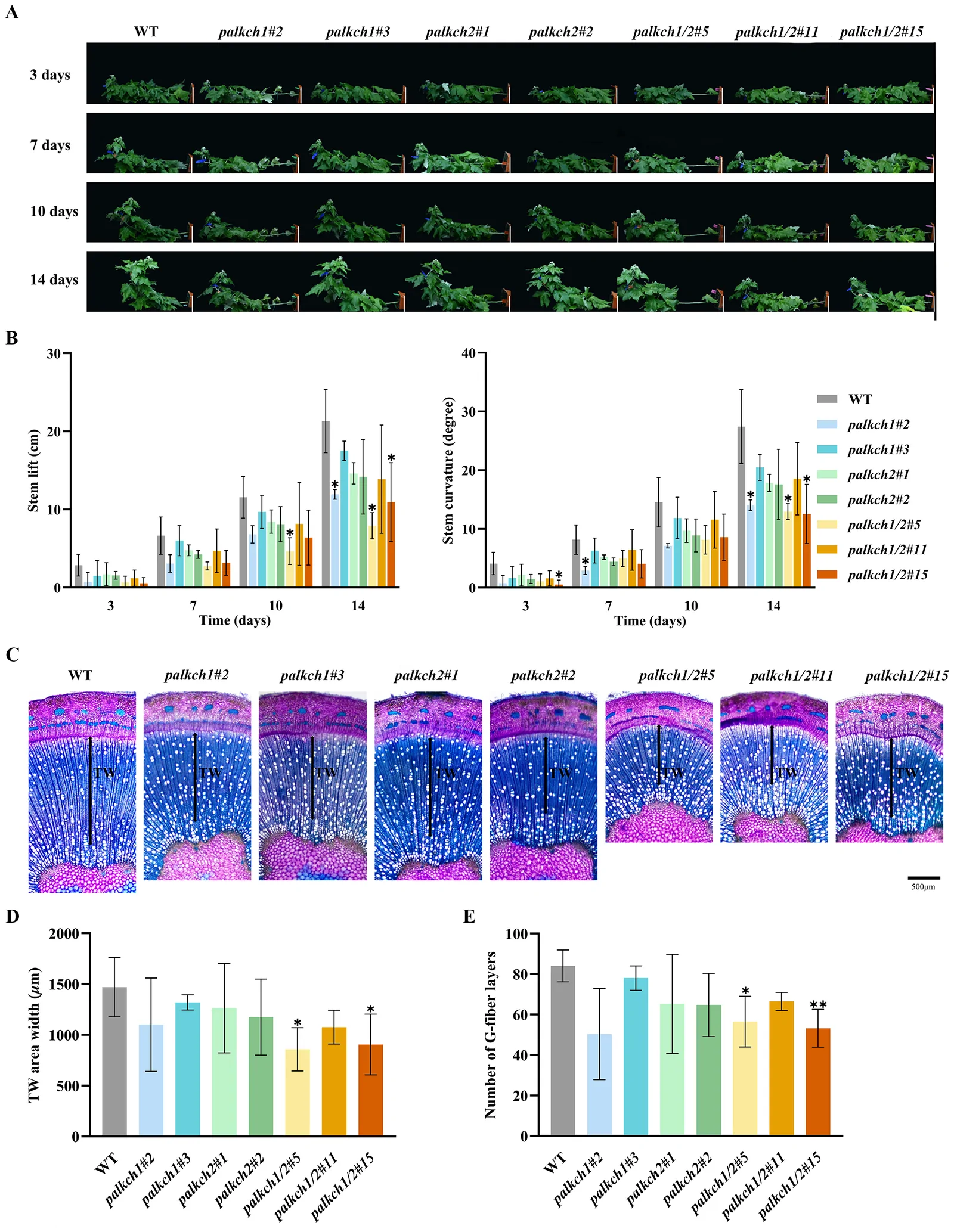
Gravitropic response and tension wood (TW) formation in wild-type (WT) and mutant plants. **(A)** Time-course images of the gravitropic response in WT, *palkch1*, *palkch2*, and *palkch1/2* plants over 14 days of gravistimulation. Scale bar = 10 cm. **(B)** Quantitative analysis of stem elevation height **(cm)** and bending angle (degree) at 3, 7, 10, and 14 days post-bending induction. Data are presented as mean ± SD (n = 4 independent biological replicates). Asterisks (*) indicate statistically significant differences between the indicated line and the wild-type (WT) control at the corresponding time point (* *P* < 0.05, Two-way ANOVA followed by Dunnett’s multiple comparisons test). **(C)** Cross-sections taken after 14 days of treatment from the 8th internode that had been marked prior to gravistimulation, showing TW formation in WT and mutant lines. Scale bar = 500 *μ*m. **(D, E)** Quantitative measurements of maximum TW region width (*μ*m) and number of G-layers in the corresponding sections. Data are mean ± SD (n ≥ 3 biological replicates). Asterisks indicate significant differences between the WT and each mutant line at each time point (* *P* < 0.05, ** *P* < 0.01; one-way ANOVA followed by Bonferroni’s multiple comparisons test).

We further quantified TW region width and G-layer frequency 14 days after treatment (**Figures 2C–E**). Mutant plants displayed significantly reduced TW region width and lower G-layer frequency compared with WT, and these reductions became progressively more pronounced from single to double mutants (**Figures 2C–E**).

### PalKCH1 and PalKCH2 modulate the responsiveness of wood cell wall constituents under TW stimulation

To investigate how PalKCH1 and PalKCH2 influence cell wall composition during TW formation, we measured lignin and cellulose contents in normal wood and tension wood from WT and mutant plants. In normal wood, lignin and cellulose contents did not differ significantly between WT and any mutant line (**Figures 3A, B**), indicating that PalKCH1 and PalKCH2 are not required for basal cell wall biosynthesis under normal conditions.

**Figure 3:**
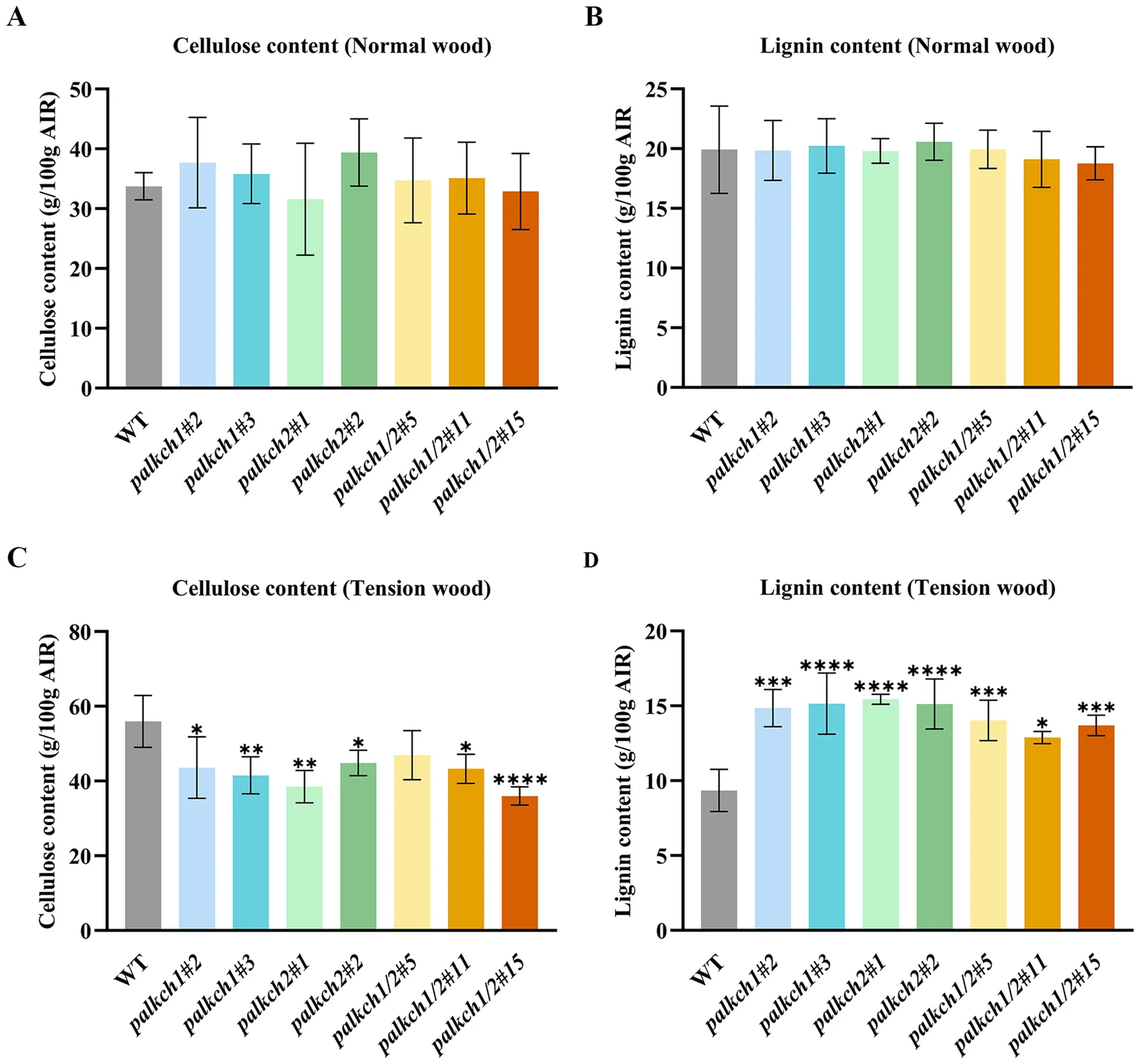
Cellulose and lignin contents in WT plants and mutant lines. **(A, B)** Cellulose **(A)** and lignin **(B)** contents under normal growth conditions. **(C, D)** Cellulose **(C)** and lignin **(D)** contents under tension wood (TW) inducing conditions. Data are presented as mean ± SD (n ≥ 3 biological replicates). Asterisks indicate significant differences relative to WT under the same condition (* *P* < 0.05, ** *P* < 0.01, *** *P* < 0.001, **** *P* < 0.0001; one-way ANOVA followed by Bonferroni’s multiple comparisons test). (Normal wood, NW; tension wood, TW).

Under TW-inducing conditions, WT plants showed the expected changes: lignin decreased significantly and cellulose increased markedly (**Figures 3C, D**). All mutant lines followed the same overall trend, but the magnitude of these changes was significantly smaller than in WT. Single and double mutants did not differ significantly from each other (**Figures 3A–D**), pointing to a threshold effect in G-layer cellulose deposition rather than a simple additive contribution of PalKCH1 and PalKCH2.

### Transcriptomic profiling identifies downstream pathways associated with PalKCH1/2 deficiency in TW

To assess the reliability of our transcriptomic data, we first performed sample correlation analysis and principal component analysis (PCA). The Pearson correlation matrix showed high consistency among biological replicates (**Supplementary Figure S7A**), and PCA clearly separated the four sample types (**Supplementary Figure S7B**), confirming that the sequencing data were of high quality.

We then applied Mfuzz clustering to genome-wide expression profiles, which identified four distinct expression patterns (**Supplementary Figure S8**). Cluster 4 contained genes with stronger induction in WT_TW than in WT_NW or the *palkch1/2* mutant lines, matching the expected transcriptional pattern for tension wood formation.

Differential expression analysis was performed across sample groups. In WT, comparison of WT_TW and WT_NW revealed a large number of differentially expressed genes (DEGs), indicating extensive transcriptomic reprogramming during TW formation (**Supplementary Figure S9A**). The comparison between *palkch1/2*_TW and *palkch1/2*_NW also produced a significant transcriptional response in the mutant background, but the total number of DEGs was lower than in WT (**Supplementary Figure S9B**). Under normal growth conditions, the transcriptional profiles of *palkch1/2* and WT were largely similar, with few DEGs detected (**Supplementary Figure S9C**). Upon TW induction, however, the number of DEGs between the two genotypes increased substantially (**Supplementary Figure S9D**).

To identify biological processes associated with PalKCH1 and PalKCH2 during TW development, we performed Gene Ontology (GO) and KEGG pathway enrichment analyses on three gene sets: Cluster 4 genes, genes upregulated in WT_TW versus WT_NW, and genes downregulated in *palkch1/2*_TW versus WT_TW. These gene sets showed substantial overlap in enriched biological processes and pathways. GO analysis identified significant enrichment of terms related to plant cell wall biosynthesis, cytoskeleton transport, cell cycle and nuclear division, and auxin and ethylene signaling (**Figure 4**). KEGG analysis yielded consistent results (**Supplementary Figure S10**).

**Figure 4:**
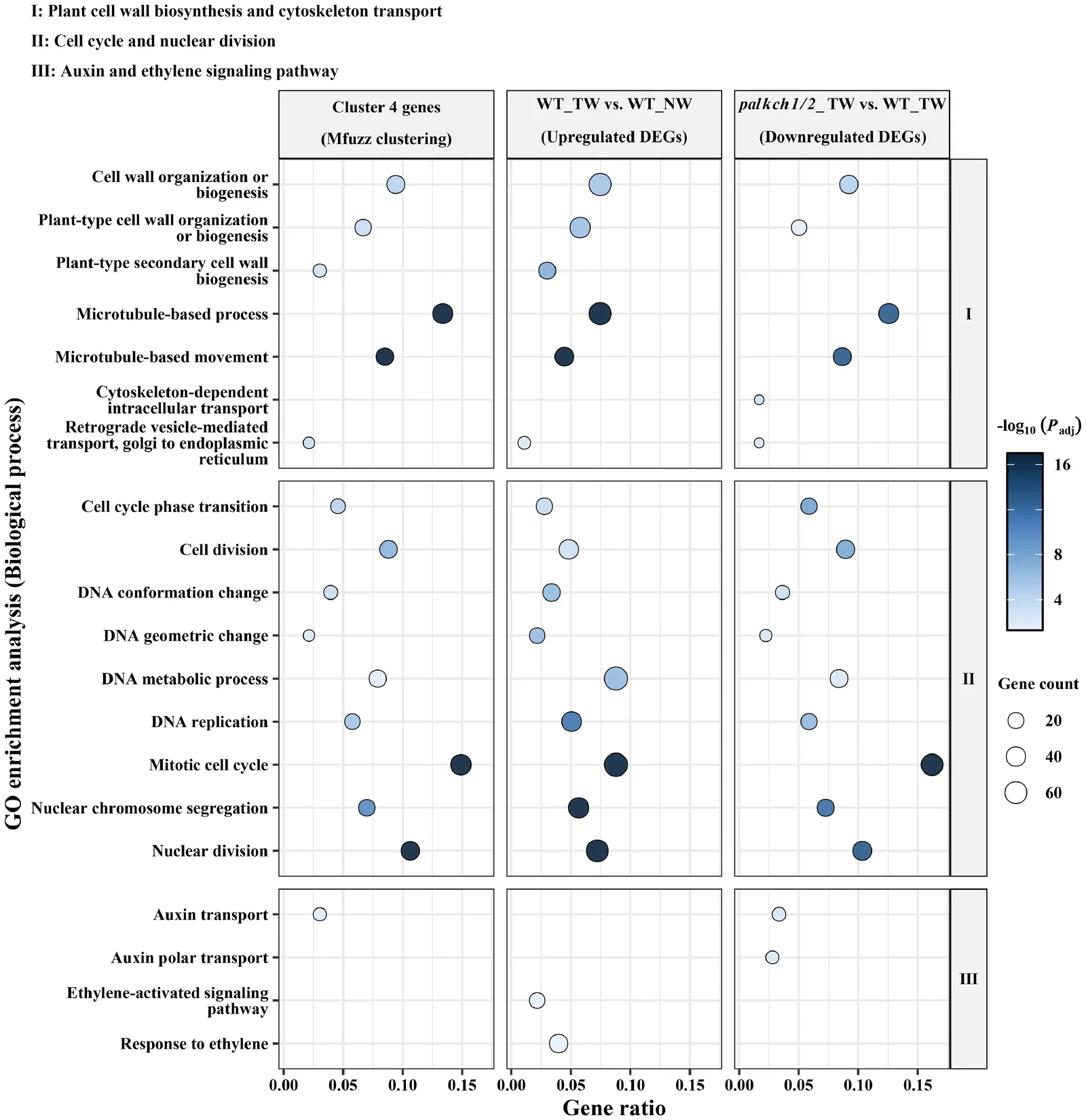
Gene Ontology (GO) enrichment analysis (Biological processes) of key candidate genes involved in tension wood formation. The three columns correspond to three gene sets: Cluster 4 identified by Mfuzz, genes upregulated in WT_TW vs. WT_NW, and genes downregulated in *palkch1/2*_TW vs. WT_TW. Enriched terms are grouped by functional classification (labels on the right). Bubble color indicates the enrichment *P*_adj_, and bubble size represents the number of enriched genes. (Normal wood, NW; tension wood, TW).

Venn diagram analysis across the three key datasets identified 425 candidate genes specifically associated with tension wood formation (**Supplementary Figure S11**). Given the recurrent enrichment of cell division-related terms, we added the cambium regulator *PalWOX4A* (Dai et al., 2023) to the candidate set. Together with genes related to cytoskeleton organization, vesicle trafficking, and cell wall remodeling from the Venn set, these were combined into a single expression heatmap (**Figure 5**). Based on their functional annotations, these DEGs were g grouped into three functional modules: cytoskeleton and vesicle trafficking, cell wall remodeling, and cambial cell cycle/proliferation. Genes in all three modules were strongly upregulated in WT_TW, but this induction was markedly reduced in the *palkch1/2* mutants (**Figure 5**).

**Figure 5:**
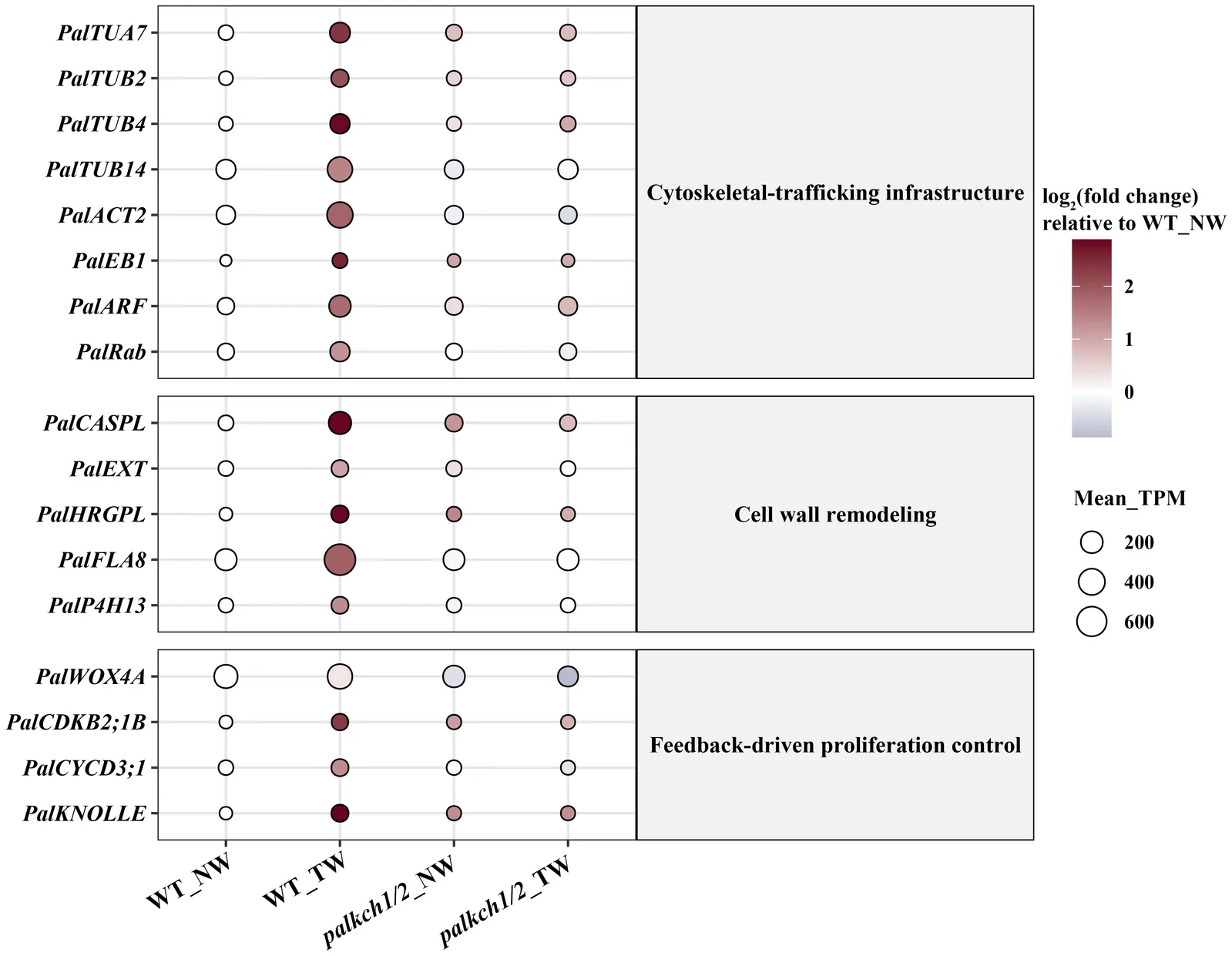
Expression profiles of key candidate genes involved in tension wood formation. These genes are grouped into three functional modules: “Cytoskeleton–vesicle trafficking infrastructure”, “Cell wall remodeling”, and “Feedback-driven proliferation control”. Bubble size represents the mean expression level (mean TPM), and bubble color indicates the log_2_(fold change) relative to WT_NW. All expression data were obtained from transcriptomic analysis of four sample groups: WT_NW, WT_TW, *palkch1/2*_NW, and *palkch1/2*_TW. (Normal wood, NW; tension wood, TW).

To validate key structural candidates from the transcriptomic analysis, we focused on genes within the cytoskeleton and vesicle trafficking module. RNA-seq showed significant upregulation of the tubulin-related gene *PalTUA7*, the actin-related gene *PalACT2*, and the vesicle trafficking-related gene *PalARF* (Pang et al., 2025) in WT_TW (**Figure 6A**), which was confirmed by RT-qPCR (**Figure 6B**). In *palkch1/2* mutants, these genes were still induced in tension wood, but their transcript levels were significantly lower than in WT. This pattern is consistent with the attenuated expression of genes related to cytoskeleton organization and vesicle trafficking identified in the enrichment analysis. Similarly, the cell wall modification factor *PalFLA8* (MacMillan et al., 2010, 2015) and the cell cycle regulators *PalWOX4A* (Dai et al., 2023) and *PalCDKB2;1B* (Wang and Pan, 2025) were upregulated in WT_TW but less induced in the mutants (**Supplementary Figure S12**).

**Figure 6:**
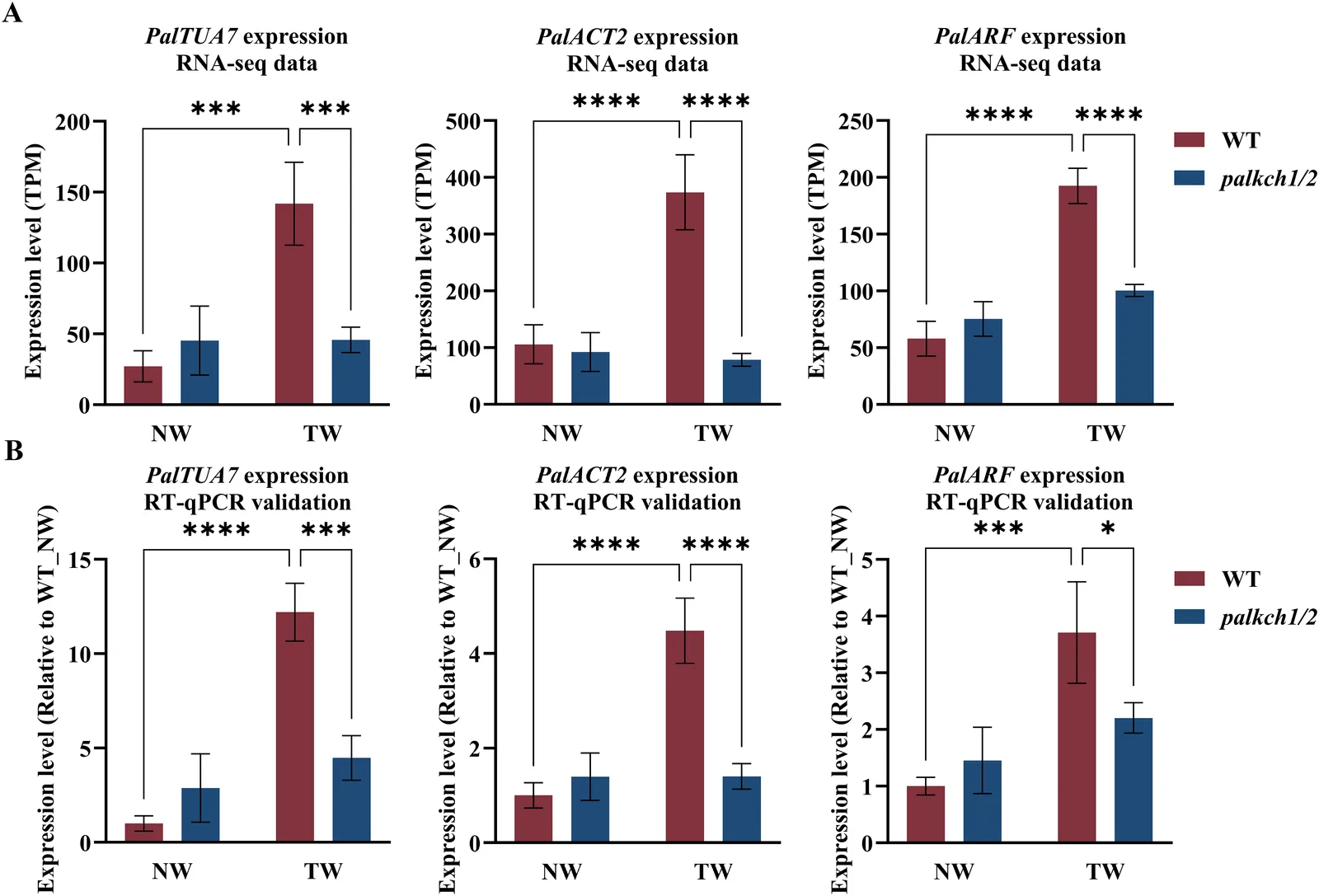
Expression analysis and validation of key candidate genes in the “Cytoskeleton–vesicle trafficking infrastructure” module during tension wood formation. **(A)** RNA-seq-derived transcript levels (TPM) of *PalTUA7*, *PalACT2*, and *PalARF* in in WT and *palkch1/2* mutants under normal wood (NW) and tension wood (TW) conditions based on RNA-seq data. **(B)** RT-qPCR validation of the corresponding genes in the same sample sets. Data are mean ± SD (n = 3 biological replicates). Statistical significance was assessed relative to WT_TW (* *P* < 0.05, *** *P* < 0.001, **** *P* < 0.0001; two-way ANOVA followed by Fisher’s LSD test).

## Discussion

Our results indicate that the Group I KCH kinesins PalKCH1 and PalKCH2 are required for normal tension wood (TW) formation in *Populus*. We propose that these kinesins mediate cytoskeletal coordination during G-layer biogenesis. Cortical microtubules and actin filaments are well established regulators of cellulose deposition, yet the molecular linkers that couple these two systems during specialized secondary wall differentiation, such as G-layer assembly, have remained elusive. PalKCH1 and PalKCH2 each contain a motor domain that binds microtubules and a CH domain that binds actin (**Figure 1A**), suggesting they could act as structural bridges between the two cytoskeletal systems. Consistently, genes suppressed in the *palkch1/2* double mutant were enriched for functions related to cytoskeletal organization, vesicle trafficking, and cell wall remodeling. Together with the reduced G-layer formation, these data support a model in which PalKCH1 and PalKCH2 coordinate interactions between microtubules and microfilaments to facilitate targeted secretion of cell wall materials during G-layer assembly (**Figure 7**). This extends the known function of plant KCH kinesins as cytoskeletal integrators (Klotz and Nick, 2012; Li et al., 2021) to a developmentally specialized cell wall differentiation program.

**Figure 7:**
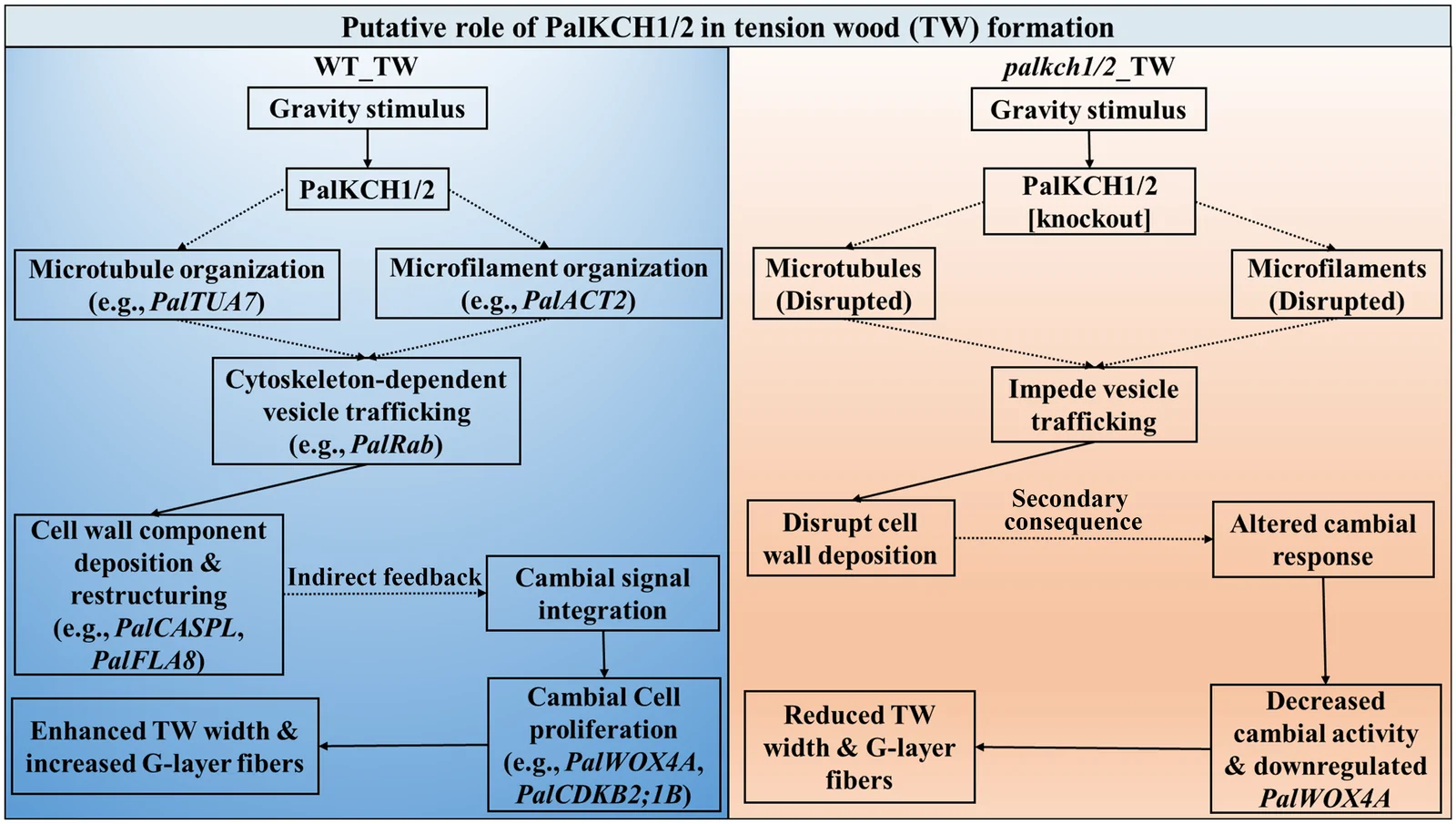
Proposed conceptual model for the putative role of PalKCH1/2 in tension wood (TW) formation in *Populus alba* var. *pyramidalis*. Under gravity stimulus, PalKCH1/2 coordinate microtubule and microfilament organization in developing fiber cells to facilitate cytoskeleton-dependent vesicle trafficking, which promotes cell wall component deposition and restructuring (e.g., *PalCASPL*, *PalFLA8*). The structural integrity of the cell wall provides an indirect feedback signal to integrate cambial responses, enhancing cambial cell proliferation (e.g., *PalWOX4A*, *PalCDKB2;1B*) and ultimately leading to increased TW width and G-layer fibers in wild-type trees (WT_TW, left panel). Conversely, knockout of *PalKCH1/2* (*palkch1/2*_TW, right panel) disrupts cytoskeletal organization and impedes vesicle trafficking, resulting in defective cell wall deposition. As a secondary consequence, the compromised G-layer structural integrity triggers altered cambial signaling, dampening cambial activity and downregulating *PalWOX4A*, which ultimately reduces TW width and G-layer formation. Solid arrows represent relationships, phenotypic features, or transcriptomic responses directly demonstrated in this study; dashed arrows indicate proposed or hypothesized downstream pathways, indirect feedback, or secondary consequences.

### Feedback regulation between fiber localized PalKCH1/2 function and cambial activity

Transcriptomic data at the single cell level showed that *PtrKCH1* and *PtrKCH2* are predominantly expressed in fiber cells rather than in the cambial region (**Supplementary Figures S3A, B**). This implies that these genes do not directly control cambial division but instead function during secondary cell wall deposition in developing fibers. However, transcriptomic analysis revealed that genes downregulated in *palkch1/2*_TW relative to WT_TW were significantly enriched for cell cycle and cell division pathways (**Figure 4**), and the cambial marker gene *PalWOX4A* was also downregulated (**Figure 5**; **Supplementary Figure S12**).

We interpret this apparent contradiction as an indirect feedback effect: loss of PalKCH1/2 first disrupts G-layer integrity in fibers, and this structural impairment triggers a signal that is not cell autonomous and suppresses cambial proliferation. Consistent with this idea, several genes associated with cell wall integrity (CWI) were significantly downregulated in *palkch1/2* tension wood, including *PalCASPL*, *PalEXT*, *PalHRGPL*, *PalFLA8*, and *PalP4H13* (**Figure 5**). These genes contribute to cell wall scaffolding and structural modification (MacMillan et al., 2015; Velasquez et al., 2015; Johnson et al., 2017; Zhang et al., 2024). Their altered expression suggests that G-layer defects caused by PalKCH1/2 deficiency activate an endogenous surveillance pathway that monitors cell wall integrity in xylem cells.

In *Populus*, impaired secondary cell wall integrity can regulate cambial activity in a manner that is not cell autonomous. By expressing xylanase under a promoter specific to the secondary wall to disrupt xylan in developing xylem, Sivan et al. (2025) observed altered cambial differentiation, reduced xylem formation, and reprogramming of the transcriptional network associated with the cambium. These results indicate that cell wall damage signals are transmitted from fiber and vessel cells to the cambium. Such feedback likely involves a CWI perception hormone cascade: receptor kinases of the CrRLK1L family (e.g., THE1) sense mechanical and chemical perturbations at the interface between the cell wall and the plasma membrane and subsequently modulate growth and cell division through ABA and JA signaling (Vaahtera et al., 2019). In this context, the downregulation of genes related to the cell wall in *palkch1/2* mutants (**Figure 5**) reflects compromised G-layer integrity. This signal is probably transmitted to the cambial region through a similar cascade, ultimately downregulating *PalWOX4A* and suppressing cell cycle pathways in the mutant TW (**Figures 4**, **5**; **Supplementary Figure S12**). Importantly, these transcriptomic changes are likely downstream responses to compromised wall integrity rather than direct transcriptional targets of PalKCH1/2.

We cannot exclude an alternative scenario in which G-layer defects alter the mechanical environment surrounding fiber cells, leading to an abnormal distribution of tissue tension. Cambial cells might then adjust their division activity by sensing changes in mechanical forces rather than receiving a specific chemical signal. In the *Arabidopsis* shoot apical meristem, mechanical stress influences organogenesis by regulating microtubule reorganization and growth anisotropy (Hamant et al., 2008). Analogously, in *Populus* TW, a defective G-layer could reduce the contractile stress generated during fiber maturation; the cambium could sense this reduction and respond by lowering division activity. These two mechanisms, chemical signaling and mechanosensing, are not mutually exclusive and could operate in parallel. Complementation assays specific to fibers will be necessary to distinguish between cell autonomous and non cell autonomous functions.

### Changes in cell wall components and phenotypic severity

TW is typically rich in cellulose and poor in lignin, a chemical signature largely attributable to the differentiation of the G-layer in fiber cells. We measured cellulose and lignin contents in the TW regions of WT and mutant lines after gravistimulation. All genotypes showed increased cellulose and decreased lignin upon TW formation; however, the magnitude of these changes was significantly smaller in the mutants than in the WT (**Figures 3C, D**). This indicates that knocking out PalKCH1/2 does not alter the fundamental biochemical character of TW. Although TW development is compromised in the mutants, the tissue that does form still follows the same cellulose-rich, lignin-poor trajectory.

Interestingly, whereas tension wood width decreased progressively from WT to single to double mutants (**Figures 2C, D**), cellulose and lignin contents did not differ significantly between the single and double mutants (**Figures 3C, D**). We attribute this discrepancy to different sensitivity thresholds of PalKCH1/2 for chemical composition at the single cell level versus development at the tissue scale. At the individual fiber level, reduced PalKCH1/2 dosage may lower the cellulose deposition capacity of the G-layer to a baseline, such that cell wall composition becomes similar in single and double mutants, a threshold effect. At the tissue level, however, the number of fibers successfully recruited into the G-layer differentiation program, or the duration of their synthetic activity, appears to decrease further as more functional alleles are lost, leading to progressive narrowing of the TW region. Thus, PalKCH1/2 influences both G-layer composition at the cellular level and the spatial extent of TW formation at the tissue scale.

A technical caveat is that our measurements represent bulk averages of the entire tension wood region. Because TW width was significantly reduced in the double mutants, the sampled tissue inevitably contained a higher proportion of adjacent normal wood, which would lower the apparent cellulose content and raise the lignin content. Thus, the attenuated chemical shifts in the mutants may partly reflect this sampling effect. However, the single and double mutants showed comparable cellulose and lignin contents despite their clearly different TW widths. This similarity is difficult to explain by dilution alone, lending further support to a threshold effect. Chemical analyses at the single cell or individual fiber level using microspectroscopy or laser capture microdissection (LCM) will help resolve this issue.

Furthermore, the cellulose microfibril angle (MFA) is a critical indicator of G-layer quality in tension wood. In typical G-layers, cellulose microfibrils are oriented nearly parallel to the fiber axis, enabling substantial contractile stress generation (Clair et al., 2011). Given that PalKCH1/2 proteins contain domains that bind microtubules and that microtubule orientation guides cellulose synthase complex (CSC) trajectories and subsequent microfibril alignment, deleting these genes may compromise not only total cellulose content but also microfibril alignment. Even if total cellulose content remains relatively stable, altered microfibril orientation could significantly diminish the mechanical performance of the G-layer. This hypothesis remains to be tested by direct MFA measurements using X ray diffraction or polarized light microscopy.

### Alternative mechanisms and perspectives

While we attribute these defects primarily to impaired cytoskeletal coordination, alternative developmental or physiological mechanisms should be considered. The reduced tension wood width and lower G-layer frequency could partly stem from delayed fiber differentiation kinetics or subtle shifts in the cell fate commitment of cambial derivatives under gravity stimulus. Furthermore, given the broad role of kinesins in intracellular transport, PalKCH1/2 deficiency might broadly constrain vesicle trafficking or induce metabolic stress, indirectly impairing cell wall material delivery. Although the specific enrichment of PalKCH1/2 in tension wood fibers points toward a specialized function, cumulative effects from developmental delay or cellular stress cannot be entirely excluded. Transcriptomic analyses with high temporal resolution combined with single cell trajectory analysis will help clarify these possibilities.

Several lines of investigation are essential to further validate and extend the proposed model. First, genetic complementation of the *palkch1/2* double mutant using constructs driven by a promoter specific to fibers will confirm their cell autonomous roles and exclude background mutation effects. Second, live cell imaging of functional fluorescently tagged PalKCH1/2 together with cytoskeletal markers will establish their subcellular localization and dynamics in developing fibers. Third, biochemical identification of PalKCH1/2 interacting partners via coimmunoprecipitation, pull down, or proximity labeling assays will clarify whether they directly bridge microtubules and actin filaments *in vivo*. Finally, detailed structural characterization of G-layer ultrastructure and cytoskeletal organization using transmission electron microscopy and high resolution imaging will provide physical correlates for the observed functional defects.

In summary, this study proposes that the *Populus* KCH kinesins PalKCH1 and PalKCH2 are required for proper G-layer formation during tension wood development. These proteins likely facilitate oriented cellulose deposition by coupling microtubule and actin filament dynamics, while their deficiency indirectly suppresses cambial activity through cell wall integrity feedback signaling. Although direct cytological visualization and biochemical interaction assays are still needed, these findings provide a framework for understanding how cytoskeletal integration and tissue level feedback regulate specialized secondary cell wall biogenesis.

## Data Availability

RNA‐seq data have been deposited in BIG Submission (GSA ID: CRA046124).
