# Supplementary material for: PalKCH1 and PalKCH2 are required for tension wood formation and gelatinous layer deposition in Populus
Source: Front Plant Sci. 2026 Sep 21;17:1942251. doi: 10.3389/fpls.2026.1942251 (PMC13635195; doi:10.3389/fpls.2026.1942251)
Supplement: Supplementary file 1 [file DataSheet1.docx]

**Fig. S1. Selection and validation of reference genes during tension wood (TW) formation.** (A) Expression stability of five candidate reference genes across different induction stages (0, 3, and 7 days), determined by TPM values from transcriptome analysis. (B) Raw Ct values of candidate genes in pooled samples. (C) Absolute ΔCt values between TW and control (Normal wood, NW) samples. The dashed line indicates the stability threshold (|ΔCt| = 0.5), demonstrating that *PalSF* exhibits the highest stability during TW induction. (D) Verification of *PalSF* expression robustness across multiple biological replicates. Error bars represent the mean ± standard deviation (SD) of three biological replicates. (E) Robustness analysis of *PalSF* expression across WT and palkch1/2 genotypes subjected to TW and NW treatments. Error bars represent the mean ± SD (n = 3 biological replicates).

**Fig. S2. Expression kinetics and spatiotemporal expression profiles of the *PtrKCH* gene family.** (A) Expression patterns of *PtrKCH1*–*PtrKCH11* under tension wood (TW) induction. B0, B3, and B5 represent 0, 3, and 5 days of mechanical bending in *Populus trichocarpa*, respectively. (B) Refined quantitative analysis of highly expressed *PtrKCH1*–*PtrKCH4* members from (A) at different stages of TW induction. Data are presented as mean ± SD (n = 3 biological replicates, **P* < 0.05, ***P* < 0.01, two-way ANOVA). (C) Transcriptional responses of *PtrKCH1*–*PtrKCH11* during TW formation (Tension_0 day to Tension_7 day). (D) Refined quantitative analysis of highly expressed *PtrKCH1*–*PtrKCH4* members from (C) during TW induction. Data are presented as mean ± SD (n = 3 biological replicates, **P* < 0.05, ***P* < 0.01, two-way ANOVA). (E) Spatiotemporal expression heatmap of the *PtrKCH* gene family in normal and TW sections, based on laser capture microdissection (LCM) datasets. (F) log_2_(fold change) and -log_10_(*P*_adj_) values of *PtrKCH* genes significantly upregulated in fiber development regions (Regions 1–3). Red dashed lines denote the statistical significance threshold.

**Fig. S3. Single-cell expression profiles of the *PtrKCH* gene family in *Populus trichocarpa* xylem.** Violin plots showing the normalized expression distribution of (A) *PtrKCH1*, (B) *PtrKCH2*, (C) *PtrKCH3*, and (D) *PtrKCH4* across distinct cell clusters in the xylem. Abbreviations: FuOs, fusiform organizers (purple); FuEPs, fusiform early precursors (green); FuIPs, fusiform intermediate precursors (brown); F, fibers (red); V, vessel elements (blue); ROs, ray organizers (orange); RPs, ray precursors (yellow); R, ray parenchyma (pink).

**Fig. S4. Tissue-specific expression and tension wood responsiveness of *PalKCH3* and *PalKCH4.*** (A, B) Tissue-specific expression and TW response of *PalKCH3*. (C, D) Tissue-specific expression and TW response of *PalKCH4*. Data are presented as mean ± SD (n = 3 biological replicates). Asterisks indicate statistical significance (**P* < 0.05, *****P* < 0.0001; A, C one‑way ANOVA with Dunnett's post-hoc test; and B, D unpaired *t*-test with Welch's correction). (Normal wood, NW; tension wood, TW).

**Fig. S5. Genotypic characterization of CRISPR/Cas9-generated knockout lines.** (A) CRISPR/Cas9-mediated editing of *PalKCH1* in *palkch1* single mutant lines (*palkch1#2* and *palkch1#3*). Sequences display the sgRNA target site (boxed), PAM motif (red), and identified deletions (dashed lines). (B) Mutation analysis of *PalKCH2* in *palkch2* single mutant lines (*palkch2#1* and *palkch2#2*). (C) Targeted mutation identification in *PalKCH1* and *PalKCH2* loci for *palkch1/2* double mutant lines (*palkch1/2#5*, *palkch1/2#11*, and *palkch1/2#15*). Distinct colors and boxes denote different sgRNA target sites. Mutant alleles are indicated by “+x bp” or “-x bp”. (D) Schematic representation of truncated protein structures. Full-length PalKCH1/2 proteins comprise calponin-homology (CH) and motor domains, with predicted truncations in mutant lines illustrating the loss of these functional domains.

**Fig. S6. Phenotypic analysis of wild-type (WT) and mutant lines under normal growth conditions**. (A) Representative morphology of WT, *palkch1* single mutants (*palkch1#2* and *palkch1#3*), *palkch2* single mutants (*palkch2#1* and *palkch2#2*), and *palkch1/2* double mutants (*palkch1/2#5*, *palkch1/2#11*, and *palkch1/2#15*). Scale bar = 10 cm. (B) Cross-sections of the xylem at the 15th internode of WT and mutant lines. Scale bar = 500 *μ*m. (C, D) Quantitative measurements of xylem width (*μ*m) and xylem area (mm²) at the 15th internode. Data are expressed as mean ± SD (n ≥ 3 biological replicates). No significant differences were observed between WT and any mutant line (one-way ANOVA followed by Bonferroni's multiple comparisons test, *P* > 0.05).

**Fig. S7. Quality assessment and reproducibility analysis of transcriptomic data.** (A) Hierarchical clustering analysis based on Pearson correlation coefficients of gene expression levels among biological replicates. The color scale indicates the strength of correlation. (B) Principal component analysis (PCA) of genome-wide expression profiles. Samples are labeled by genotype (WT and *palkch1/2*) and treatment. Each data point represents an independent biological replicate (n = 3 biological replicates). PC1 (30.9%) and PC2 (19.6%) represent the first and second major sources of variance, respectively. (Normal wood, NW; tension wood, TW).

**Fig. S8. Mfuzz clustering analysis of gene expression profiles across four conditions: WT_NW, WT_TW, *palkch1/2*_NW, and *palkch1/2*_TW.** (Normal wood, NW; tension wood, TW)

**Fig. S9. Volcano plot analysis of differentially expressed genes (DEGs) across different comparison groups.** (A–D) Volcano plots illustrating the distribution of DEGs, where the x-axis represents log_2_(fold change) and the y-axis represents -log_10_(*P*_adj_). Comparison groups: (A) WT_TW vs. WT_NW; (B) *palkch1/2*_TW vs. palkch1/2_NW; (C) *palkch1/2*_NW vs. WT_NW; (D) *palkch1/2*_TW vs. WT_TW. Red circles represent significantly upregulated genes, blue squares represent significantly downregulated genes, and gray triangles represent genes with no significant difference. Vertical dashed lines indicate the fold change thresholds (-1 and 1). (Normal wood, NW; tension wood, TW)

**Fig.S10. Kyoto Encyclopedia of Genes and Genomes (KEGG) pathway enrichment analysis of key candidate genes involved in tension wood formation.** The three columns correspond to three gene sets: Cluster 4 identified by Mfuzz, genes upregulated in WT_TW vs. WT_NW, and genes downregulated in *palkch1/2*_TW vs. WT_TW. Enriched terms are grouped by functional classification (labels on the right). Bubble color indicates the enrichment *P*_adj_, and bubble size represents the number of enriched genes. (Normal wood, NW; tension wood, TW).

**Fig. S11. Venn diagram analysis for the identification of candidate genes involved in tension wood formation.** The Venn diagram illustrates the overlap among four gene datasets: (a) genes in Mfuzz Cluster 4 (highly expressed in WT_TW); (b) DEGs upregulated in WT_TW compared to WT_NW; (c) DEGs downregulated in *palkch1/2*_TW compared to WT_TW; and (d) DEGs downregulated in *palkch1/2*_NW compared to WT_NW. The red number (425) represents the intersection of datasets (a), (b), and (c), with overlapping genes from dataset (d) excluded. (Normal wood, NW; tension wood, TW).

**Fig. S12. Expression analysis and validation of key candidate genes involved in "Cell wall remodeling" and "Feedback-driven proliferation control" during tension wood formation.** (A) Transcript levels (TPM) of *PalFLA8*, *PalWOX4A*, and *PalCDKB2;1B* in WT and *palkch1/2* mutants under NW and TW conditions, based on RNA-seq data. (B) RT-qPCR validation of the expression levels for these three key genes in corresponding samples. Data are presented as mean ± SD of three biological replicates. Statistical significance was assessed relative to WT_TW (n = 3 biological replicates, ***P* < 0.01, ****P* < 0.001, *****P* < 0.0001; two-way ANOVA followed by Fisher's LSD test). (Normal wood, NW; tension wood, TW).
